# A role for the canonical nuclear factor-κB pathway in coupling neurotrophin-induced differential survival of developing spiral ganglion neurons

**DOI:** 10.3389/fncel.2013.00242

**Published:** 2013-11-28

**Authors:** Renaud Vandenbosch, Eva Chocholova, Pierre A. Robe, Yiqiao Wang, Cécile Lambert, Gustave Moonen, François Lallemend, Brigitte Malgrange, Saïda Hadjab

**Affiliations:** ^1^Groupe Interdisciplinaire de Génoprotéomique Appliquée-Neurosciences, Developmental Neurobiology Unit, University of LiègeLiège, Belgium; ^2^Department of Human Genetics, University of LiègeLiège, Belgium; ^3^Groupe Interdisciplinaire de Génoprotéomique Appliquée-Research Center, University of LiègeLiège, Belgium; ^4^Department of Neuroscience, Karolinska InstituteStockholm, Sweden; ^5^Bone and Cartilage Research Unit, Institute of Pathology, Centre Hospitalier Universitaire du Sart-TilmanLiège, Belgium; ^6^Department of Neurology, Centre Hospitalier Universitaire du Sart TilmanLiège, Belgium

**Keywords:** spiral ganglion neurons, neurotrophins, neuronal survival, development, NF-κB, cochlea

## Abstract

Neurotrophins are key players of neural development by controlling cell death programs. However, the signaling pathways that mediate their selective responses in different populations of neurons remain unclear. In the mammalian cochlea, sensory neurons differentiate perinatally into type I and II populations both expressing TrkB and TrkC, which bind respectively brain-derived neurotrophic factor (BDNF) and neurotrophin-3 (NT3). How these two neuronal populations respond differentially to these two neurotrophins remains unknown. Here, we report in rat the segregation of the nuclear factor-κB (NFκB) subunit p65 specifically within the type II population postnatally. Using dissociated cultures of embryonic and postnatal spiral ganglion neurons, we observed a specific requirement of NFκB for BDNF but not NT3-dependent neuronal survival during a particular postnatal time window that corresponds to a period of neuronal cell death and hair cell innervation refinement in the developing cochlea. Consistently, postnatal *p65* knockout mice showed a specific decreased number in type II spiral ganglion neurons. Taken together, these results identify NFκB as a type II neuron-specific factor that participates in the selective survival effects of BDNF and NT3 signaling on developing spiral ganglion neurons.

## INTRODUCTION

In the developing nervous system, the selectivity of the neurotrophins for the survival of particular populations of neurons during development depends not only on the specific spatiotemporal pattern of expression of the ligand in the target tissue but also of its corresponding Trk receptors in the neurons. However, while Trk receptors are structurally similar, their specific binding can lead to the activation of different signaling pathways with distinct impacts on target innervation and neuronal survival *in vivo* (Postigo et al., 2002; Nikoletopoulou et al., 2010). Although significant progress has been made in our understanding of the specificity of the downstream effectors different Trk receptors are using, the molecular basis supporting these differences remains not well understood.

Spiral ganglion neurons (SGNs) constitute the first relay of the acoustic neuroanatomical ascending pathway between the sensory receptors – the inner and outer hair cells (IHCs/OHCs) of the organ of Corti – of the mammalian cochlea and the central nervous system (Nayagam et al., 2011). SGNs are divided in two types: type I neurons innervating specifically the IHCs, represent the principal encoder of the auditory signal and account for about 90% of the neuronal population while the type II neurons, representing the remaining 10%, contact the OHCs and are thought to provide an integrated afferent feedback loop and to amplify both cochlear sensitivity and frequency discrimination (Geleoc and Holt, 2003). The development of SGNs starts at mid gestation and is completed during the two first postnatal weeks in rodents. Among neurotrophins, brain-derived neurotrophic factor (BDNF) and neurotrophin-3 (NT3) through their respective receptors, TrkB and TrkC, have been shown to play a central role in regulating the development of SGNs (Fritzsch et al., 2005; Defourny et al., 2011; Yang et al., 2011). Indeed, they are expressed and secreted by the developing otic sensory epithelium and act on neuronal TrkB and TrkC to control their survival and the growth of their projections towards the hair cells (Farinas et al., 2001; Defourny et al., 2011). As such, mice with knockouts of the NT3 or the BDNF genes (or of their specific receptors) have shown a marked reduction, of about 80–90% (NT3-null) and 10% (BDNF-null), in SGN number (Farinas et al., 1994; Ernfors et al., 1995; Tessarollo et al., 1997; Wiechers et al., 1999). This differential sensitivity suggested a specific survival function of NT3 and BDNF for type I and II SGNs, respectively. Although subsequent studies have shown that the specificity of neurotrophin-dependent survival for the two types of SGNs may also depend on the baso-apical location within the cochlea (Fritzsch et al., 1997; Farinas et al., 2001), the analysis of a specific TrkB mutant, which lacks part of the TrkB intracellular signaling pathway and survives until adulthood, confirmed this essential role of the BDNF-TrkB signaling in controlling the survival of type II SGNs (Schimmang et al., 2003). This targeted survival promoting action of BDNF was also observed *in vitro* where treatment of postnatal SG explants with BDNF rescued essentially the type II population in the absence of organ of Corti-derived endogenous survival-promoting factors (Barclay et al., 2011). However, TrkB and TrkC are expressed in all SGNs, regardless of their type, and both specific ligands are located within the hair cells during development (Farinas et al., 2001), which is not consistent with a differential localization of neurotrophin support but rather suggests that the two types of SGNs are endowed with distinct molecular signaling machineries that are responsible for the preferential survival promoting action of NT3 and BDNF on the type I and II SGNs.

Nuclear factor-κB (NFκB) is an inducible transcription factor that acts as a dimer whose prototype is p50/p65, with p65 containing the transactivation domain. p50/p65 is abundant in numerous cell types, including neurons, in which it is involved in a wide range of functions including differentiation and survival (Meffert and Baltimore, 2005). In its silent state, p50/p65 is sequestered in the cytoplasm by the inhibitory proteins IκBs (mostly IκBα). Upon activation of an IκB kinase complex, IκBα is phosphorylated and releases p50/p65 dimers that rapidly translocate into the nucleus and act on gene expression (Hayden and Ghosh, 2012). In the nervous system, NFκB is widely expressed and is activated by a variety of neurotrophic factors, including neurotrophins (Gutierrez and Davies, 2011). In addition, activation of NFκB prevents neuronal apoptosis in various cell culture and *in vivo* models (Mattson et al., 2000). In the present study, we studied the dynamic expression of NFκB in the developing rat SGNs. We observed that p65 is first expressed in all SGNs before birth. During postnatal development, p65 is exclusively present within type II SGNs, an expression profile that remains unchanged afterwards. Interestingly, during this early postnatal period, we found a marked p65 activity in type II neurons. At the same time, we observed that the survival-promoting function of BDNF, but not of NT3, on cultured SGNs depends on NFκB activity. Finally, the analysis of *p65* knockout mice revealed a large decrease in the number of type II SGNs. Altogether, our results propose the p65 subunit of the transcription factor NFκB as a type II neuron-specific key molecular factor which distinguishes between BDNF- and NT3-dependent survival signaling in SGNs.

## MATERIALS AND METHODS

### MOUSE LINES

To examine the role of p65 in SGNs, viable *p65*^-^^/^^-^ mice were generated on a *Tnfr1*-deficient background as described previously (Meffert et al., 2003) and maintained as heterozygous. Mice were group-housed in the animal facility of the University of Liege under standard conditions with food and water ad libitum and were maintained on a 12-h light/dark cycle. All animals were taken care of in accordance with the declaration of Helsinki and following the guidelines of the Belgian ministry of agriculture in agreement with EC laboratory animal care and use regulation.

### PRIMARY CULTURES OF SGNs

Experiments were performed on Wistar rat SGNs from embryonic day (E) 18 to postnatal day (P) 8 rats. Rat species was chosen here as their cochlea is not or incompletely ossified at the ages analyzed, and SG from rat cochlea can be easily isolated. Rats were euthanized and decapitated. Mandibles were removed, bullae exposed and temporal bones excised and transferred into Petri dishes containing phosphate buffered saline (PBS) supplemented with glucose (6 g/l). With the aid of a dissecting microscope and watchmaker’s forceps, the cochlea was isolated and its SG excised. SG were incubated for 40 min at 37°C in a papain-DNAse solution (1.5 ml of papain at 1 mg/ml, 0.5 ml of DNAse at 0.1%; 20 SG/2 ml solution). Enzymatic activity was terminated by adding ovalbumin (0.5 ml of a 10 mg/ml solution). The explants were then washed with DMEM supplemented with N1 additives (Bottenstein and Sato, 1979) and glucose (6 g/l, final concentration). Mechanical dissociation of the SG tissue was achieved by trituration with a siliconised Pasteur pipette. For cell survival experiments, the resulting ganglion cell suspension was seeded in 96-well plates (Nunc, Rockside, Denmark) previously sequentially coated with poly-L-ornithine (0.1 ng/ml in 15 mM borate buffer) for 1 h and laminin (10 μg/ml in PBS) for 2 h at 37°C. Cells were maintained at 37°C in a humidified atmosphere of 5% CO2/95% air. Neurotrophins were purchased from Peprotech (Rocky Hill, NJ, USA), sulfasalazine, from Sigma (St Louis, MO, USA) and SN-50, from Calbiochem (San Diego, CA, USA).

### IMMUNOSTAININGS

For immunohistochemistry, rat cochleae were fixed in 4% paraformaldehyde at 4°C overnight, decalcified (from P5 onward) in EDTA solution and transferred into a solution of 30% sucrose in PBS for an additional overnight incubation at 4°C. Cochleae were then embedded in Tissue Tek (Sakura, the Netherlands) and frozen at -80°C. Frozen 10–20 μm sections were prepared using a 2800 Frigocut cryostat (Reichert-Jung, Cambridge Instruments, Germany). Neuronal cultures or cryostat sections were fixed with 4% paraformaldehyde (10 min at 20°C). The preparations were then incubated overnight at 4°C with primary antibodies, i.e., anti-betaIII-tubulin (1/1500; clone TUJ1, Babco, USA), a specific marker for neuronal lineage (Fanarraga et al., 1999), and anti-p50 (1/50, Delta Biolabs), anti-p65 and anti-IκBα (1/50, Santa Cruz Inc., CA, USA). Three washes in PBS were performed before incubation for 1 h at room temperature, with a secondary antibody conjugated to either peroxidase (Dako, Glostrup, Denmark), FITC (fluorescein isothiocyanate) or TRITC (tetramethylrhodamine B isothiocyanate) (Jackson Immunoresearch Laboratories Inc., West Grove, PA, USA) fluorophores. After three rinses in PBS, the immunofluorescent preparations were mounted on microscope slides, mounted in Vectashield (Vector, Burlingame, CA, USA) and imaged using an Olympus FV1000 laser scanning confocal microscope. Peroxidase-labeled cells were revealed using the DAKO Liquid DAB+ kit, a high sensitivity substrate-chromogen system (DAKO Diagnostics N.V./S.A., Heverlee, Belgium), and then examined with an inverted microscope (Zeiss, Axiovert 100, Germany). Negative controls were carried out by omission of the primary antibody or by using the blocking peptide for each antibody (data not shown).

### NEURONAL SURVIVAL AND NEURITOGENESIS DETERMINATION

At the end of the culture period, cells were fixed and immunostained for TUJ1 and peroxidase secondary antibody as described above. Criteria used to determine neuronal viability were (1) TUJ1 positivity, with a large labeled cytoplasm, and (2) an absence of nuclear pyknosis. The measure of the extent of neuritogenesis was achieved as previously described (Lallemend et al., 2005a). Briefly, the number of neurons bearing neuritic processes that were of a defined length of at least three neuronal cell body diameters were counted. Neurites were counted only if they had an obvious attachment to the neuronal soma. The neuritic index was calculated as the ratio of neurons bearing neurites of the defined length or greater per total number of neurons.

### WESTERN BLOT ANALYSIS

A total of about 20 SG (representing one sample) from rat embryos or pups (E18-P10) were lysed on ice using 100 μl of lysis buffer (1% triton X-100, 150 mM NaCl, 50 mM Tris-HCl, pH 7.4, 2 mM DTT, 1 mM sodium orthovanadate and protease inhibitors cocktail; Roche). After 30 min, the lysate was cleared of insoluble debris by centrifugation for 10 min at 11,000 *g*. The supernatant was collected, and the protein concentration was determined using the Bradford method. Protein lysates (30 μg of proteins) were then mixed with an equal volume of gel loading buffer (glycerol 20%, SDS 4%, Tris 100 mM, β-mercaptoethanol 5% and bromophenol blue) before being boiled for 3 min. After boiling, proteins were loaded onto a 10% SDS-PAGE. The proteins were then transferred on a polyvinylidene difluoride membrane (Amersham, Roosendaal, the Netherlands) by semi-dry electroblotting in transfer buffer (Glycine 192 mM, Tris 25 mM and methanol 20%). Blots were then blocked for 1 h at room temperature in blocking buffer [0.2% I-BLOCK (TROPIX, Bedford, MA, USA) diluted in TBS supplemented with 0.05% Tween 20 (Bio-Rad, Nazareth, Belgium), TTBS]. The primary antibodies were incubated for 1 h at room temperature in the blocking buffer. Peroxidase-conjugated monoclonal anti-rabbit antibodies (clone RG-16, Sigma Aldrich, 1:3000) were incubated 1 h at room temperature. Blots were then washed extensively and developed by using enhanced chemoluminescence (Pierce, Aalst, Belgium).

### RIBOPROBE SYNTHESIS

pRc/CMV plasmids containing anti-sense and sense primers for amplification of *p65* and *p50* were kindly provided by Dr. S. Memet from Pasteur Institute in Paris. Plasmids were cloned in bacteria mach1TM-T1R (Invitrogen) and isolated using QIAprep spin miniprep Kit from Qiagen (Germany). The complementary strands for sense and for antisense riboprobes were transcribed from either Sp6 or T7 RNA polymerases and labeled using rNTP mix containing digoxigenin-labeled rNTP purshached from Roche Diagnostics (Germany).

### IN SITU HYBRIDIZATION

Riboprobes were diluted to appropriate concentrations in the hybridization buffer (Amresco) containing 50% formamide and were denatured for 10 min at 65°C. Riboprobes were applied to sections for overnight hybridization in a 50% formamide chamber at 55°C. Sections were washed twice in 0.1 × SSC (300 mM sodium chloride and 30 mM sodium citrate, pH 7.0) at 55°C for 30 min. After a brief wash in Tris buffer (0.1 M Tris-HCl and 0.15 M sodium chloride, pH 7.5), sections were blocked in Tris buffer containing 0.5% blocking reagent (catalog #1096176; Boehringer Mannheim) and 0.3% Triton X-100 and incubated with anti-digoxigenin antibody conjugated to alkaline phosphatase (1:750; Boehringer Mannheim). Sections were exposed to staining solution containing nitroblue tetrazolium salt and 5-bromo-4-chloro-3-indolyl phosphate for up to 20 h and viewed using an Olympus AX70 microscope.

### ELECTROMOBILITY SHIFT ASSAY

Nuclear protein extracts were obtained and processed for electrophoretic mobility shift assay as described previously (Robe et al., 2004).

### STATISTICAL ANALYSIS

Statistical analyses were performed by one-way ANOVA using a GraphPad Prism program (GraphPad, San Diego, CA, USA). Individual comparisons were performed using Dunnett’s post hoc tests. Data were expressed as the mean ± S.D. and results were considered statistically significant if *P* < 0.05 for both the one-way ANOVA and the Dunnett’s test.

## RESULTS

### DEVELOPMENTAL DYNAMIC OF p65, p50 AND IκBα EXPRESSION IN THE SG

**FIGURE 1:**
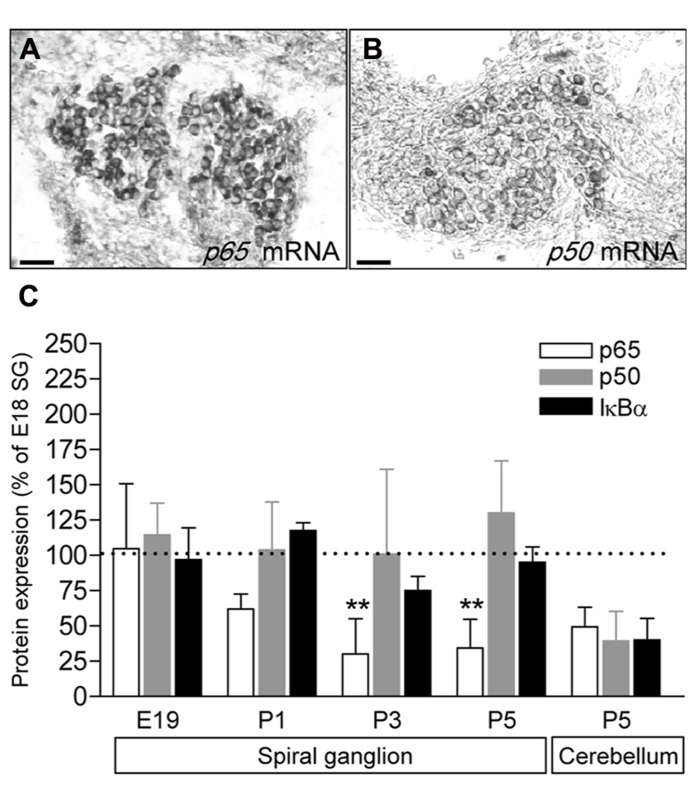
**Developmental expression of p65, p50 and IκBα in rat SG.** **(A,B)** *p65* and *p50* mRNA expression in P5 rat SG sections assessed by *in situ *hybridization. Scale bar: 50 μm. **(C)** SG proteins were extracted from E19 to P9 rat cochlea or P5 cerebellum and analyzed by western-blot for the expression of the p65, p50, IκBα and actin proteins using specific antibodies. Densitometric analyzes were performed and results were expressed as the ratio of targeted proteins *versus* actin (P9 data are not shown and are similar to P5). Results are expressed as a percentage of the ratio quantified at E19. Note the large decrease in p65 expression postnatally (**P < 0.01, compare E19 to P3 or P5). The graph represents the mean of 3–6 experiments.

To study the expression of the NFκB complex in the SG, we first analyzed the expression of *p65* and *p50* transcripts by in situ hybridization. Data revealed that at P5 (similar results from E18 to 1 year old, data not shown), mRNA for both *p50* and *p65* are restricted to the neuronal population (**Figures 1A,B**). We then analyzed the general expression pattern of p65, p50 and of IκBα in whole protein extracts of SG from E18, E19, P1 and P5 animals. As illustrated in **Figure 1C**, a semi-quantitative analysis (data are normalized to the values obtained at E18) showed that the level of expression of the three proteins remained unchanged during late embryonic development. However, while the expression of p50 and IκBα was stable from late embryonic to postnatal stages, the expression of p65 protein dramatically decreased at P1 and remained at low level during the following days (**Figure 1C**).

### p65 IS SELECTIVELY EXPRESSED IN TYPE II NEURONS IN POSTNATAL AND ADULT COCHLEA

**FIGURE 2:**
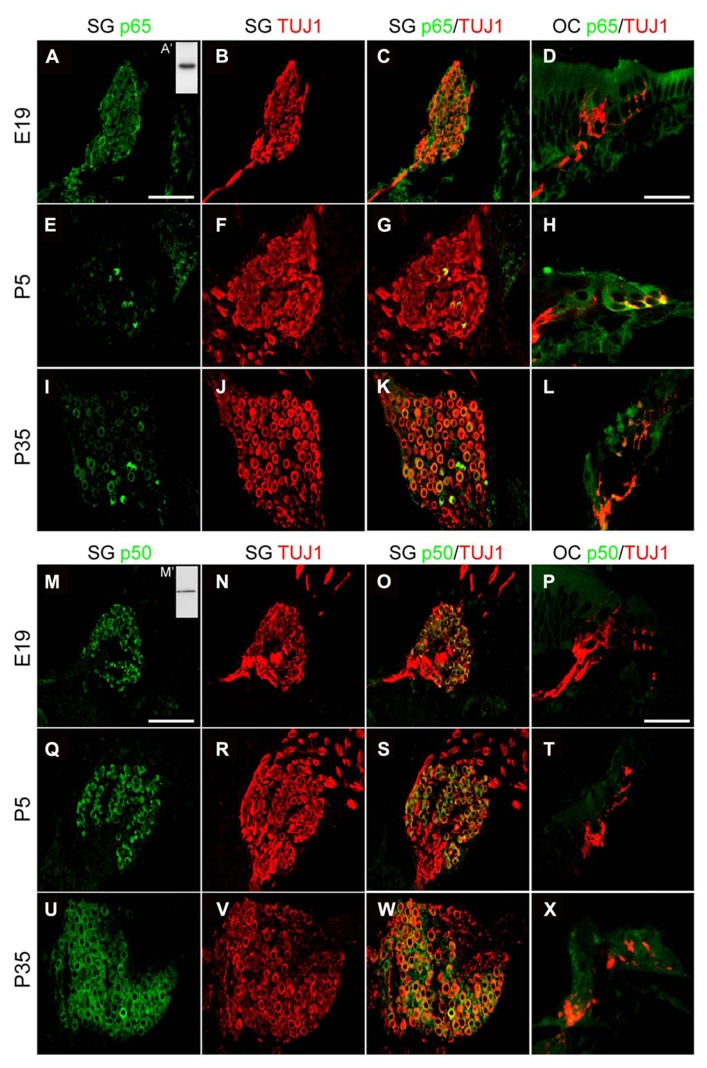
**Distribution of p65 and p50 proteins in the developing cochea.** **(A–L)** Representative confocal images for the expression of p65 subunit (green) and the neuronal marker TUJ1 (red) on rat SG (**A–C**, **E–G**, **I–K**; scale bar: 100 μm) and corresponding organs of Corti (OC; **D,H,L**; scale bar: 40 μm) from E19, P5 and P35 rat cochlear sections. Inset in **A** (A**’**), represents immunoblotting for p65 from P5 rat SG total protein extract which reveals a specific single band staining at the expected molecular weight. **(M–X)** Representative confocal images for the expression of p50 subunit (green) and the neuronal marker TUJ1 (red) on rat SG (**M**–**O**, **Q**–**R**, **U**–**W**; scale bar: 100 μm) and OC (**P,T,X**; scale bar: 40 μm) from E19, P5 and P35 rat cochlear sections. Inset in **M** (M**’**), represents immunoblotting for p50 from P5 rat SG total protein extract which reveals a specific single band staining at the expected molecular weight. On the same immunoblot, the specific band corresponding to p105 is not shown.

**FIGURE 3:**
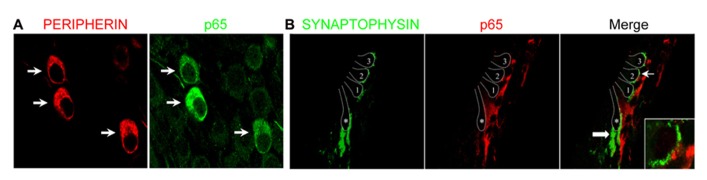
**Preferential expression of p65 in postnatal type II SGNs.** **(A)** Double immunostaining for p65 and peripherin on SG sections from P7 rats showing restriction of p65 expression in peripherin^+^ type II neurons (arrows). **(B)** Double immunostaining for synaptophysin (efferent marker) and for p65 on P7 rat cochlear section at the level of the organ of Corti showing the absence of colocalization (arrow and inset). 1–3: the three rows of outer hair cells; asterisk: the inner hair cell; thin arrow: the position of the higher magnification represented in the inset; thick arrow: efferent nerve endings under IHC.

The marked decrease of p65 expression in whole SG observed during the first postnatal week may indicate a general downregulation of p65 or a selective expression within a specific neuronal population. To address this issue, we examined the temporal and spatial distribution of p65 expression on cochlear sections of different developmental stages using antibodies against p65, βIII-tubulin (a general neuronal marker; Fanarraga et al., 1999) and peripherin, a type III intermediate filament protein that serves as a reliable marker for type II neurons at postnatal and adult stages (Hafidi, 1998; Lallemend et al., 2007). At late embryonic stage, we observed that p65 is widely expressed in the whole SG, staining most if not all sensory neurons (**Figures 2A–C**). In contrast, after birth, p65 immunostaining could be visualized in only a few neurons (**Figures 2E–G**). This pattern of expression was maintained at adult stage, where the small size of the p65 immunopositive neurons indicates that they are type II neurons (**Figures 2I–K**). A close analysis of the immunostaining at the level of the organ of Corti revealed a clear staining below the OHCs, but only during the early postnatal period (**Figures 2D,H,L**). A further analysis of both peripherin and p65 expression confirmed that these p65 positive neurons were type II neurons (**Figure 3A**). OHCs innervation consists of type II afferents from the SG and efferents that convey information from the brain to the cochlea (Knipper et al., 1996; Wiechers et al., 1999). To distinguish between these two types of fibers and confirm that the p65 positive projections below the OHCs are only type II afferents, we labeled postnatal cochlea sections for both p65 and synaptophysin, a specific maker for presynaptic terminations that stain efferents below the hair cells (Knipper et al., 1995). Synaptophysin positive nerve endings were seen below each OHC, but never colocalized with the p65 positive projections, indicating that these last projections are exclusively type II afferents (**Figure 3B**). Later during development, around P11 when hearing starts in rat, the expression of p65 could no longer be seen within the SG (data not shown). It reappeared however, at around P15-16 in type II neuronal cell bodies, but not in their projections, an expression profile that was maintained at adult stage (**Figure 2L**).

In contrast to p65, the expression of p50 was expressed in all SGNs, but not in their projections, at all stages analyzed (**Figures 2M–X**).

### TRANSITORY *IN VIVO* ACTIVITY OF NFκB DURING EARLY POSTNATAL PERIOD

**FIGURE 4:**
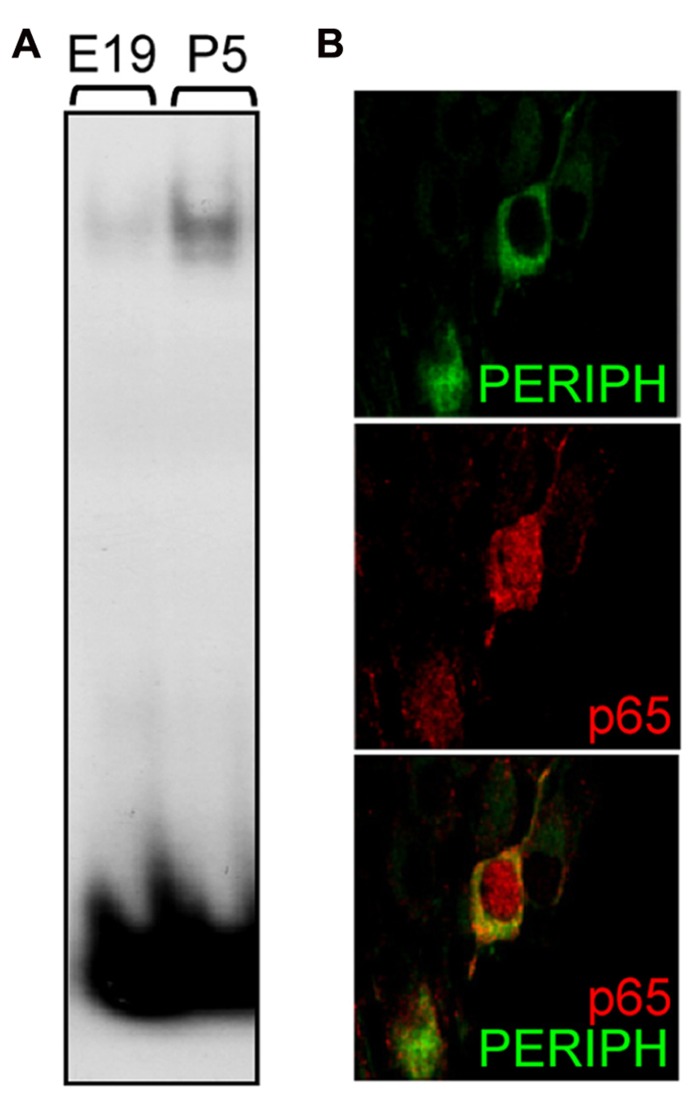
**Postnatal activation of p65 in type II SGNs.** **(A)** Nuclear extracts from E19 and P5 SG were analyzed by EMSA using a specific κB probe. Note the large increase in DNA-protein complex observed postnatally. **(B)** Double immunostaining for peripherin (green) and for p65 (red) on P5 rat SG section showing nuclear staining for p65 in a type II neuron. Scale bar: 10 μm.

The above results suggest the participation of NFκB in biological processes that regulate the early postnatal development of type II neurons. To assess whether NFκB particularly is activated during that period, we studied the DNA-binding activity of p65 in SG at different stages. Using a gel shift assay, we observed that the activity of p65 subunit in SG nuclear extracts was virtually absent at late embryonic stage, appeared at P1 (data not shown) and markedly increased at P5 (**Figure 4A**; NFκB activity, E19: 1 ± 0.13, P5: 7.27 ± 1.61; *n *= 4, *P* < 0.001, *t*-test, normalized to E19). These results were confirmed by analyzing the subcellular localization of p65 in type II neurons at postnatal stages. Indeed, one hallmark of NFκB activity is its translocation within the nucleus, which could be observed in many type II neurons at P4-5, as illustrated in **Figure 4B**. Immunolabeling for p65 was never observed in type I neurons after birth.

### BDNF-INDUCED SURVIVAL OF SGNS REQUIRES NFκB SIGNALING DURING EARLY POSTNATAL PERIOD

**FIGURE 5:**
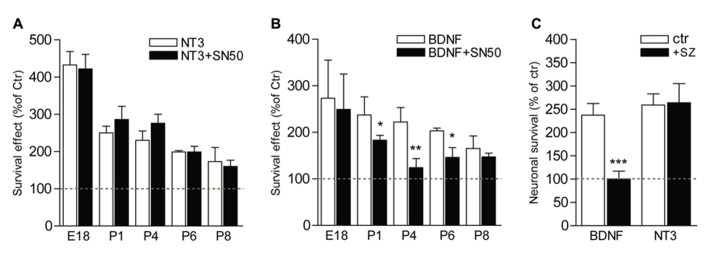
**NFκB is necessary for BDNF but not NT3-induced survival of postnatal SGNs.** **(A–C)** SGNs were cultured for 24 h with BDNF or NT3 (each at 20 ng/ml) and in the presence of specific inhibitors of the NFκB signaling, SN50 (5 μM) **(A,B**) or sulfasalazine (1 mM, sz; P4, **C**). Note the absence of effect of the inhibitors on survival function of NT3 while they dramatically decrease the survival promoting action of BDNF (*n *= 3–8; *P < 0.05, **P < 0.01, ***P < 0.001; compared to neurotrophin alone).

The postnatal period is characterized by a refinement of the innervation of the organ of Corti by SGNs, and a concomitant loss of neurons within the type II population, which depends on BDNF for their survival (Barclay et al., 2011). To investigate the implication of NFκB in the signaling of the trophic support mediated by neurotrophins on SGNs during this critical period of development, SGNs from different stages of development, from embryonic (E18 and E19) or postnatal (P1, P2, P4, P6, and P8) animals, were cultured for 24 h in the presence of BDNF or NT3 together with specific inhibitors of the NFκB pathway, i.e., SN50 that blocks nuclear translocation of NFκB (Lin et al., 1995) and sulfasalazine (SZ) that interferes with the phosphorylation of IκBα (Wahl et al., 1998). Both neurotrophins were shown to significantly increase survival of SGNs (*P *< 0.01 at P8 and *P *< 0.001 at E18-P6, compared to control) from embryonic or postnatal animals, as previously shown (Malgrange et al., 1996; Mou et al., 1997, 1998). Strikingly, while inhibitors of NFκB did not affect the survival-promoting activity of NT3 at all stages analyzed (*P *> 0.05, **Figures 5A,C**), the trophic action of BDNF was specifically and only affected during the early postnatal period of development, from P1 to P6, with a maximum of inhibition observed at P4 (**Figures 5B,C**; *P *> 0.05, BDNF+SN50 compared to control). In addition to their survival action, neurotrophins have been shown to affect axonal growth both *in vivo* and *in vitro* (Defourny et al., 2011). Interestingly, inhibitors of NFκB did not change the BDNF-induced axon growth of SGNs from P4 animals (*P* > 0.05), suggesting a specific requirement of NFκB signaling for survival but not general axon growth promoted by BDNF.**

### LOSS OF PERIPHERIN^+^ SGNS IN THE ABSENCE OF *P*65 *IN VIVO*

**FIGURE 6:**
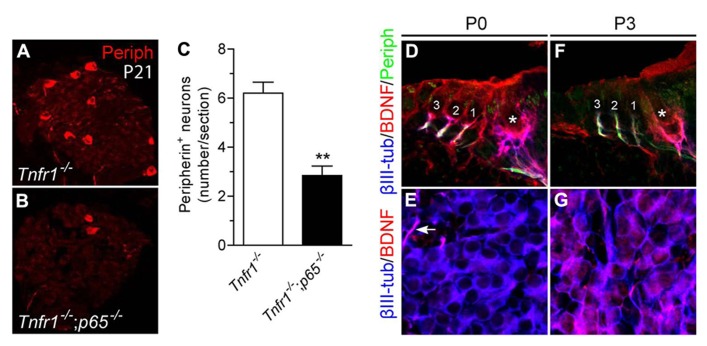
**Loss of type II SGNs in *p65*-deficient mice.** **(A,B)** Immunostaining for peripherin on SG sections from *Tnfr1*^-^^/^^-^ **(A)** and *Tnfr1*^-/-^; *p65*^-^^/^^-^ mice **(B)**, showing dramatic loss of type II population in the absence of *p65*. **(C)** Quantification of **(A,B)** (*n *= 3; **P < 0.01). **(D–G)** Immunostaining for βIII-tubulin, BDNF and peripherin on sections of organ of Corti **(D,F)** or SG **(E,G)** from P0 **(D,E)** and P3 **(F,G)** mouse cochlea. Note expression of BDNF in the OHC (1 to 3) and IHC (asterisk) at P0 **(D)** but not at P3 **(F)**. Also, while BDNF expression is observed in afferent endings of IHC and OHC at both P0 **(D)** and P3 **(F)**, it is hardly seen in the soma of SGNs at P0 (**E**, arrow points to a positive afferent), but upregulated there at P3.

To examine the requirement for p65 during development of type II neurons* in vivo*, we changed the model system and analyzed null mutant mice for *p65*. Since *p65*^-^^/^^-^ mice die at E14–15 from massive liver apoptosis mediated through TNFR1 signaling (Beg et al., 1995; Rosenfeld et al., 2000), we generated mice that were deficient in both *p65* and *Tnfr1* and analyzed neuronal phenotype in cochlea of young adult animals (P21). Interestingly, although the total number of SGNs (i.e., βIII-tubulin^+^ cells per section) showed no significant difference between *Tnfr1*^-^^/^^-^ and *Tnfr1*^-^^/^^-^; *p65*^-^^/^^-^ mice (*Tnfr1*^-^^/^^-^; *p65*^+^^/^^+^: 92.3 ± 14.4; *Tnfr1*^-^^/^^-^; *p65*^-^^/^^-^: 74 ± 4.8, *n* = 3, *P* > 0.1), the number of peripherin^+^ type II neurons (which only represents around 10% of the whole neuronal population) was dramatically reduced in the absence of *p65* (**Figures 6A–C**), whereas experiments with wild-type mice yielded similar numbers as seen for *Tnfr1*^-^^/^^-^; *p65*^+^^/^^+^ (data not shown). Together with our *in vitro* analysis, these data show that BDNF through NFκB signaling is required to support a significant fraction of the type II neuronal population during early postnatal development of the cochlea.

We next sought to define the dynamic of BDNF expression during this critical postnatal period of development. At P0, BDNF was found to be expressed in the sensory epithelium and nerve endings below the hair cells while the cell bodies of the neurons were not or barely positive (**Figures 6D,E**). In contrast, at P3, while BDNF was expressed in the cell bodies of most neurons (**Figure 6G**), regardless of the type (in both peripherin positive and negative SGNs, data not shown), it was absent in hair cells (**Figure 6F**), confirming previous results (Wiechers et al., 1999) and suggesting a local (autocrine and/or paracrine) function of BDNF in sustaining survival of type II population during the postnatal period of development.

## DISCUSSION

Although the assignment of NT3 for the survival of type I SGNs during development is still unclear, the trophic function of BDNF on type II neurons has been largely documented, both *in vitro* and *in vivo* (Ernfors et al., 1995; Wiechers et al., 1999; Schimmang et al., 2003; Barclay et al., 2011). However, BDNF is expressed in both IHCs and OHCs during early development and its high affinity receptor TrkB, in all SGNs, raising the question to know what mechanisms may mediate the differential responsiveness of type I and II neurons to NT3 and BDNF. Here, we show that p65 is selectively expressed in type II neurons postnatally and that NFκB activity is required specifically for BDNF- but not NT3-dependent survival of postnatal SGNs *in vitro*. Consistently, *p65*^-^^/^^-^ mice display large deficits in the survival of type II SGNs *in vivo*. Thus, p65 represents the first identified transcription factor that selectively regulates the survival of type II SGNs during development.

SGNs develop from the neurogenic compartment of the otic vesicle during mid-embryonic stage in rodents (Appler and Goodrich, 2011). During the following days, they send axonal projections to hair cells in the organ of Corti and the central nervous system and progressively differentiate into type I and II neurons, which clearly distinguish perinatally (Koundakjian et al., 2007). Apart from their characteristic peripheral connections with OHCs, type II neurons can be recognized postnatally by their specific expression of peripherin (Lallemend et al., 2007). We show here that the expression of p65 is present in virtually all neurons until birth and selectively expressed at high level in the type II population thereafter. Such specificity for type II neurons is unlikely to be limited to rat species, as similar pattern of p65 expression has been previously reported in gerbils (Lang et al., 2005). Moreover, the characteristic loss of type II neurons in mice lacking *p65* strongly supports the specificity of p65 expression for type II neurons after birth also in mice.

The tight control of p65 postnatally coincides exactly with peripherin down regulation and the onset of myelin marker expression in type I neurons (Knipper et al., 1998; Lallemend et al., 2007), indicating that a specific transcriptional machinery implicating both repressor and activator functions operates simultaneously at this stage to clearly define the two neuronal phenotypes of the cochlea. Interestingly, the observation that the transcript for *p65* but not the protein is found in type I neurons after birth suggests the existence of post-translational mechanisms (e.g., neuron type-specific miRNA expression) that would ensure specific expression of p65 protein in type II neurons. Also, the fact that p65 is active only during the postnatal period while its expression is observed before birth in both neuron types implies a tight regulation of signaling pathways that would control the activation of NFκB. This could be done at the cellular levels for instance by regulating either the expression of IκB kinases, which phosphorylate and inhibit IκBs (Hayden and Ghosh, 2012), or the relative amount of p65 and p50 in the neuron, what would impact the formation of potentially transcriptionally active p65/p50 heterodimers *versus* p50 homodimers that are transcriptionally inactive or negative regulators of transcription (Hayden and Ghosh, 2012).

The early outgrowth of peripheral projections of SGNs leads at birth to an incomplete innervation pattern in the organ of Corti, where IHCs and OHCs have been shown to be innervated by both types of neurons (Huang et al., 2007). This immature configuration is corrected during the following days by a progressive pruning ultimately providing the adult-like innervation pattern where type I and II neurons contact exclusively the IHCs and OHCs, respectively (Huang et al., 2007; Defourny et al., 2011). This reorganization is accompanied by a 25% neuronal loss that mostly concerns the type II population (Rueda et al., 1987; Barclay et al., 2011) and likely participates in the elimination of type II afferents underneath the IHCs. Interestingly, early postnatal type II neurons are rescued by BDNF *in vitro* (Barclay et al., 2011). NFκB signaling has been shown to act downstream of neurotrophic factor receptors (including Trks) activation to promote neuronal survival (Maggirwar et al., 1998; Huang and Reichardt, 2003; Mincheva et al., 2011). The observation in our study of a specific requirement of p65 signaling for BDNF-induced survival of early postnatal SGNs *in vitro* and of its specific role in regulating survival of type II neurons *in vivo* strongly suggest that NFκB represents a key molecular component driving the differential survival promoting action of BDNF on type II neurons. The maintenance of p65 expression in type II SGNs in adult raises the question whether NFκB would play a similar survival function on this population under stress conditions, where neuroinflammatory factors and calcium signaling, both known to be able to activate NFκB (Meffert et al., 2003; Hayden and Ghosh, 2012), play an important role (Gale et al., 2004; Lallemend et al., 2005b; Tornabene et al., 2006; Keithley et al., 2008).

The spatio-temporal dynamic expression of p65 in SGNs observed in our study is accompanied by changes in the expression profile of BDNF in the cochlear system. Indeed, we and others have shown that BDNF is downregulated in the sensory epithelium and start to be expressed in the majority of neurons (type I and II) during the first postnatal week (Wheeler et al., 1994; Wiechers et al., 1999; present study). This downregulation of BDNF in the organ of Corti might contribute to afferent fiber retraction from the hair cells (Wheeler et al., 1994). The initiation of BDNF expression in SGNs at the same stage could then provide a transient autocrine and/or paracrine signal whereby type II neurons, deprived of organ of Corti-derived trophic signals, could survive until BDNF is again supplied by their target at later stage (Wiechers et al., 1999).

Another interesting aspect of our data is that the intracellular signaling pathway of BDNF and NT3 is clearly different and important for their survival promoting effect. Because SGNs express both TrkB and TrkC receptors (Mou et al., 1997; Farinas et al., 2001), these differences are likely operating within the same neurons. Indeed, we show here that BDNF but not NT3 uses NFκB signaling for promoting survival of postnatal SGNs. As a result, postnatal type II neurons, which upregulate p65, will be more likely to be rescued by BDNF compared to type I neurons (Barclay et al., 2011), which is consistent with our *in vivo* results showing a selective loss of type II neurons in the absence of *p65*. Moreover, our results also suggest at least *in vitro* that BDNF would utilize different signaling pathways to promote either survival, through NFκB, or general axonal growth independently of NFκB. Similar dissociation of intracellular effectors leading to distinct biological functions has been previously described *in vivo* (Postigo et al., 2002). Although interesting, this observation will require further analysis of the possible function of NFκB on the fine arborization at nerve endings, as seen for pyramidal neurons *in vitro* (Gutierrez et al., 2005).

Altogether, these results underline the importance of understanding the differences in the neuron-type specific molecular machinery which are responsible for mediating distinct biological responses to neurotrophins and that ultimately control the proper development of the peripheral nervous system.

## Conflict of Interest Statement

The authors declare that the research was conducted in the absence of any commercial or financial relationships that could be construed as a potential conflict of interest.

## AUTHOR CONTRIBUTIONS

François Lallemend, Brigitte Malgrange, and Saïda Hadjab designed and supervised the study; Renaud Vandenbosch, Eva Chocholova, Pierre A. Robe, Yiqiao Wang, Cécile Lambert, François Lallemend, and Saïda Hadjab performed experiments; Renaud Vandenbosch, Eva Chocholova, Pierre A. Robe, Yiqiao Wang, Cécile Lambert, François Lallemend, Brigitte Malgrange, and Saïda Hadjab analyzed data; Renaud Vandenbosch, Gustave Moonen, François Lallemend, Brigitte Malgrange, and Saïda Hadjab wrote or revised the manuscript, with input from co-authors; François Lallemend, Brigitte Malgrange, and Saïda Hadjab are co-senior authors.
